# Systematic review on the effects of the physical and social aspects of community pharmacy spaces on service users and staff

**DOI:** 10.1177/17579139221080608

**Published:** 2022-03-11

**Authors:** R Dhital, S Sakulwach, G Robert, C Vasilikou, J Sin

**Affiliations:** Arts and Sciences Department, University College London, 33–35 Torrington Place, London WC1E 7LA, UK; University of Reading, Reading, UK; King’s College London, London, UK; University of Reading, Reading, UK; University of London, London, UK

**Keywords:** pharmacy, community pharmacy, health spaces, health architecture, pharmacy environment

## Abstract

**Aim:**

This systematic review aimed to provide new insights into how pharmacy spaces, or the *architecture of pharmacies*, are experienced by pharmacy service users and staff. The review sought to identify environmental factors which may influence service users’ and staff participation in community-based pharmacy health services.

**Method:**

Ten databases were searched for English language publications, using a combination of search terms relating to pharmacy service users and staff; pharmacy spaces; and health and social care outcomes. Data from the final selected studies were extracted, thematically analysed using a narrative approach and the quality of each study assessed using the Integrated quality Criteria for the Review of Multiple Study designs (ICROMS).

**Results:**

80 articles reporting 80 studies published between 1994 and 2020 were identified; they were from 28 countries, involving around 3234 community pharmacies, 13,615 pharmacy service users, 5056 pharmacists and 78 pharmacy health staff. Most studies (94%) met the ICROMS minimum score, and half did not meet the mandatory quality criteria. Four themes likely to influence service users’ and staff experiences of pharmacy health services were identified: (1) privacy; (2) experience of the physical environment; (3) professional image; and (4) risk of error.

**Conclusion:**

To optimise the delivery and experience of pharmacy health services, these spaces should be made more engaging. Future applied research could focus on optimising inclusive pharmacy design features.

## Introduction

Community pharmacy has been defined as a space where medicine-related services are provided to patients by pharmacists to promote health through person-centred care.^1,2^ Community pharmacies are essential health spaces and contribute significantly to the public health globally. However, there is currently no universal accepted definition that encompasses the broad range of activities and services provided by community pharmacy.^2^ Worldwide, community pharmacies are located where people live, work and shop. Survey conducted by the International Pharmaceutical Federation (FIP) between 2020 and 2021 reported there were around 1,609,734 community pharmacies in 76 countries and territories, serving around 75% of the world’s population.^3^ Community pharmacies in many countries vary in size and type, from large urban high-street chains to small independent stores in suburban communities and rural areas.^3^

With the growing demand for public healthcare and management of long-term conditions, community pharmacies play an important role in improving economic, social and clinical outcomes for individuals and their communities.^4^ The World Health Organization supports health-promotion activities which drive the need for community pharmacy to be an accessible resource, that is open during the evenings and at weekends with no appointment required.^5^ In addition, pharmacies provide a *social space* for communities, patients and carers alike.^6^ These interactions between visitors and staff often take on a social purpose where community-related conversations are discussed alongside health topics at the pharmacy counter, the consultation room and the shop floor.

Research highlights the value of developing patient–pharmacist relationship when providing health consultations.^7^ During these encounters, pharmacy space is recognised as an emerging and vital factor to support patient and practitioner engagement,^8–10^ emphasising the need to identify how best to optimise pharmacy spaces for people using and providing these services.

However, we do not know how these spaces are experienced by pharmacy patients and staff and the possible health and social implications of this. The design of healthcare environments, or *health architecture*, for promoting good health and wellbeing is growing into an important field of enquiry.^11^ Salutogenic architecture, in other healthcare settings, predicted on the basis that space design can improve health outcomes for patients; for example, lighting, soundscape and seating area comfort can affect a user’s experience of the physical space in such a way that it positively impacts their mental wellbeing.^12^ In addition, noise has been found to increase employees’ stress and fatigue levels which can lead to medical errors.^13^ A well-designed interior layout can reduce staff fatigue and improve patient care, for example, by enabling nurses to provide rapid assistance when at-risk patients try to get out of bed.^14^ The Joint Commission on Accreditation of Healthcare Organizations found that half of the falls cases were caused by factors in the physical environment.^13^ Spaces which have not considered inclusive design can be disabling and inhibit engagement. For example, in primary healthcare settings, physical access to spaces can present barriers, especially for the disabled, and affect the quality of care and reduce their willingness to participate in treatment.^15^ The design of hospitals and cancer care centres has been part of inclusive health architecture practice for the last two decades.^16,17^ While evidence linking the design of hospital and primary care settings to the quality and outcomes of care is increasing, there is limited research on community pharmacy spaces.

Research informed by health architecture theory highlights the importance of considering both patient and employee experience of health space.^18^ Systematic reviews relating to pharmacy public health services have identified a range of perspectives and experiences; however, these have not focused on the effects of pharmacy spaces.^19,20^ A systematic review of the existing evidence is needed to examine how community pharmacy spaces are experienced and to stimulate new understanding to effectively develop community pharmacy public health services globally. The objectives of this review were to (1) identify and appraise the designs of relevant empirical studies; (2) identify and assess the environmental factors which may influence patients’ and staff participation in pharmacy health services; and (3) explore the possible health and/or social or professional implications of these.

## Methods

The review protocol was prospectively published in PROSPERO (International Prospective Register of Systematic Review).^21^ The review process followed the PRISMA (Preferred Reporting Items for Systematic Reviews and Meta-Analyses) guideline.^22^

### Data sources and search strategy

The following 10 databases were searched for studies from their inception until 31 March 2020:

1. PubMed
2. PsycINFO (via Ovid)
3. Web of Science
4. Scopus
5. ScienceDirect
6. Journal Storage (JSTOR)
7. International Bibliography of the Social Sciences (IBSS)
8. Cochrane Central Register of Controlled Trials (CENTRAL)
9. Health Technology Assessment Database (HTA)
10. Social Care Online (SCIE)

### Search terms

Search terms were developed by applying the PICOC (P﻿opulation, P﻿henomenon of Interest, Comparison, Outcome, Context) framework (Table 1).^23^ The final search strategy was informed by combining terms relating to Population (P) of pharmacy service users and pharmacy health team (including pharmacist, pharmacy technician and medicine counter assistant);^24^ Phenomena of Interest (I) covering any physical and social elements of the pharmacy space (including pharmacy layout, pharmacy counter, dispensary, consultation room and pharmacy retail area, lighting, noise and privacy); Comparison (C) included any health interventions reported; Outcome (O): were of reported experiences of the physical and social aspects of the pharmacy space, including satisfaction, engagement, attitudes, performance or health intervention outcomes; and Context (C): comprising studies conducted within any community pharmacy settings, from any country and location (e.g. chain and independent establishments). Studies based in hospital pharmacies, clinics or online were excluded. The relevant synonyms and Medical Subject Headings (MeSH) were incorporated into the final search strategy which was adjusted for each database. Reference list of the included studies and relevant systematic reviews were checked. An exemplar search strategy as used for PubMed database is presented in Supplemental Material 1.

**Table 1:** Key search terms used for the systematic review based on PICOC (Population, Phenomenon of Interest, Comparison, Outcome, Context)

Population (P): pharmacy user or pharmacy staff	Phenomena of Interest (I): pharmacy space	Comparison (C)	Outcome (O): pharmacy outcomes	Context (C): pharmacy setting
*Pharmacy user* User^a^ Service user^a^ Customer^a^ Patient^a^ Client^a^ Pharmacy staff Pharmacist^a^ Chemist^a^ Counter staff^a^ Technician^a^	Pharmacy design Interior design Evidence-based design Physical environment^a^ Social environment^a^ Architecture^a^ Workspace^a^ Space Lighting^a^ Noise^a^ Privacy Workstation People flow^a^ Safety environment^a^ Security^a^ Comfort^a^ environment^a^ Centre of built environment	Any comparator or without comparison	Perception^a^ Experience^a^ Satisfact^a^ Participat^a^ Observation^a^ Impression^a^ Emotional effect^a^ Environment effect^a^ Engagement^a^ Involve^a^ Attitude^a^ Work efficiency Performance^a^ Workflow Work productivity Teamwork	Community pharmac^a^ Pharmac^a^ Chemist^a^

a Truncation utilises root words to find variations of terms.

### Inclusion/exclusion criteria

All primary studies, of any study design published in English, relating to pharmacy users and staff experience of the community pharmacy space were selected (Table 2). We excluded nonempirical study data, that is, from textbooks, grey literature, reviews and meta-analyses.

**Table 2:** PICOC (P﻿opulation, P﻿henomenon of Interest, Comparison, Outcome, Context): inclusion and exclusion criteria

Category	Inclusion criteria	Exclusion criteria
Population (P)	– Pharmacy users of any characteristics. – All members of the pharmacy health team: pharmacists, dispensing staff, accredited checking technicians and counter staff.	Any other population
Phenomena of Interest (I)	Any environmental factors experienced by pharmacy users and staff of the community pharmacy space. Examples of environmental factors: lighting; noise; and privacy. Examples of pharmacy spaces: health counter; dispensary area; consultation room; and retail area.	Not related to the community pharmacy spaces
Comparison (C)	Any comparison, with or without controls.	Not applicable
Outcome (O)	Any outcomes relating to pharmacy users’ and staff experiences of the community pharmacy space when accessing or delivering pharmacy health services. Outcomes include the level of privacy, adequate space and professionalism.	Not applicable
Context (C)	Community pharmacy can be part of a – Supermarket – Chain store – Independent store	– Hospital pharmacy – Other clinic settings – Online

### Study selection

All search results were inputted into an Endnote library. After removing duplicates, all titles and abstracts were screened by S.S. against the inclusion and exclusion criteria. A second reviewer (R.D.) independently screened a 5% random sample of all items. This screening process was repeated for full text of all potentially eligible papers. In addition, R.D. independently reviewed a 20% random sample of the excluded full-text papers, to address the possibility of missing potentially relevant studies. Any disagreements were resolved through discussions between R.D., J.S. and S.S.

### Quality assessment

The Integrated quality Criteria for the Review of Multiple Study designs (ICROMS) tool^25^ was chosen to appraise the quality of the included quantitative, qualitative and mixed methods studies. The first step was to classify the study design for each study to select the appropriate criteria. The next step was to evaluate scores for each study, based on the specific criteria of each of the seven dimensions, as follows: (1) clear aim and justification; (2) managing bias in sampling or between groups; (3) managing bias in outcome measurement and blinding; (4) managing bias in follow-up; (5) managing bias in other study aspects; (6) analytical rigour; and (7) managing bias in reporting/ethical considerations.

Under each dimension, the specific criteria were rated on a three-point scale (2 = meets criterion, 1 = unclear and 0 = does not meet criterion). Each study was evaluated using a decision matrix comprising two components: mandatory criteria, which refers to quality criteria which must be met; and minimum score. For this review, studies of all quality criteria were included. ICROMS has no specific criteria for surveys and mixed method study designs; we therefore used the ICROMS qualitative studies criteria to rate these across the seven key dimensions.^26^ R.D. independently assessed the quality of a 10% random sample of the included studies. Any differences were discussed with R.D., J.S. and S.S. until a consensus was agreed.

### Data extraction and synthesis

We devised a data extraction table^27^ to ensure all relevant information was included to address the review questions. The data extraction table included headings relating to study characteristics; pharmacy service user characteristics (age groups and presenting health conditions); pharmacy staff (professional role); pharmacy type; study design; outcome measures used; and results. As the systematic review involved the analysis of data from different study designs, thematic synthesis was first used to identify the main, recurring and/or significant issues through all quantitative and qualitative data.^28^ This was followed by a narrative approach^29^ focusing on the key aspects of pharmacy users’ and staff experiences.

## Results

**Figure 1:**
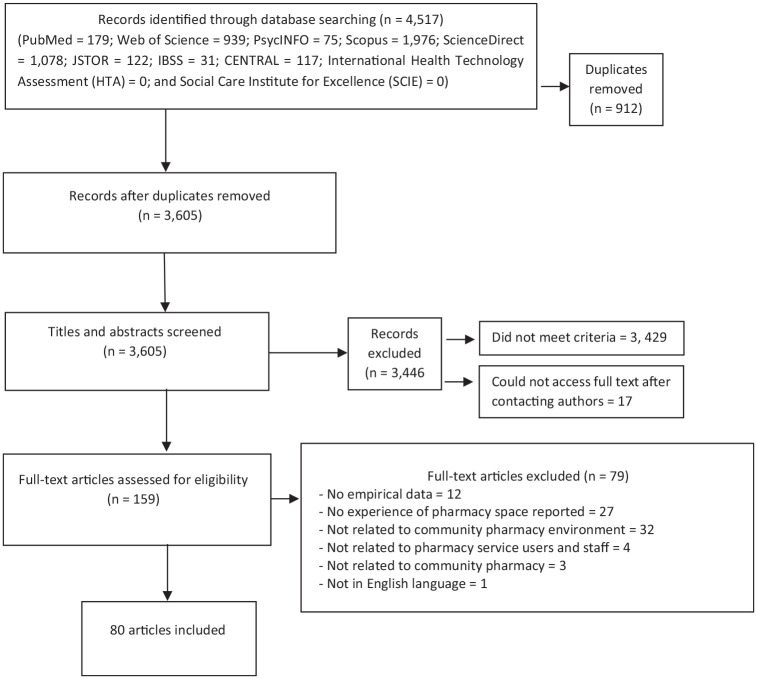
PRISMA flow diagram of the literature search and selection process

The search initially retrieved 4517 records. After screening titles and abstracts, against the inclusion criteria, 159 full-text papers were read (Figure 1, PRISMA flow diagram). From these, 80 papers reporting 80 research studies published in English between 1994 and 2020 were included.

### Overview of included studies

Most of the studies (*n* = 60) were published during 2010–2020 (Table 3). Study designs used included surveys (*n* = 40); individual interviews (*n* = 19); qualitative focus groups (*n* = 8); mixed method study (*n* = 11); nonrandomised study with a control group (‘controlled before-after’) (*n* = 1); and cohort study (*n* = 1). The 40 survey studies involved questionnaires which were administered to participants to understand their perceptions, emotions and views on a range of pharmacy health services. The mixed method studies comprised six survey/qualitative studies; two qualitative/biophotographic studies; one observational/qualitative study; one observational/survey study; and one qualitative/Delphi technique study. The nonrandomised study with a control group observed participants before and after an intervention to compare views on different pharmacy environments. The cohort study (*n* = 1) examined pharmacists’ perspectives of organisational culture in the pharmacy environment.^30^

**Table 3:** Summary characteristics of 80 included studies (*N* = number of studies or another variable as described)

Study characteristic	Study characteristic details	*N*
Study design	Survey	40
	Qualitative study	27
	Mixed methods	11
	Controlled before-after	1
	Cohort	1
Year of publication	1990–1999	2
	2000–2009	18
	2010–2019	56
	2020	4
Study continent or region	Africa	4
	Asia	6
	Australasia	16
	Europe	32
	Middle East	12
	North America	10
Pharmacy geographical areas and pharmacy type	Total community pharmacies	3234
	Area^a^- Urban	593
	- Suburban	11
	- Rural	168
	Type^a^- Independent	465
	- Chain	753
Participants’ characteristics^a^	Pharmacists	5056
	Pharmacy support staff	78
	Pharmacy service users	13,615
	Study population of focus:	
	Elderly people (aged ⩾65 years)	1 Study
	Young people (aged 12–25 years)	3 Studies
	Men’s health	1 Study
	Women’s health	1 Study
Number of studies reporting specific health conditions or services^a^	Contraception	2
	Drug and alcohol problems	6
	Gastrointestinal conditions	1
	Heart disease	3
	Intimate partner violence (IPV)	1
	Mental health	8
	Public health roles	2
	Respiratory disease	2
	Sexual health	3
	Skin conditions	1
	Smoking cessation	1
	Weight management	1

a Not all studies provided details of participants or sample size.

The 80 studies were conducted in 28 countries across six continents and region (Africa, Asia, Australasia, Europe, Middle East and North America). Of the approximate total 3234 pharmacies, 672 were reported to be in urban (*n* = 593); suburban (*n* = 11); and rural areas (*n* = 168). The definitions of ‘urban’ (city), ‘suburban’ and ‘rural’ areas were based on population densities of approximately 3000+, between 1000 and 3000 and 1000 people per square mile, respectively. ^31^ Altogether around 13,615 pharmacy service users were included as participants. Not all studies reported demographic characteristics of participants, such as age and gender. Study participants also included around 5056 pharmacists and 78 pharmacy staff (including medicine counter assistants, dispensing assistants and accuracy-checking technicians).

There were universal concerns about privacy and lack of space across the different continents. Studies focused on similar health and pharmacy practice issues irrespective of country. However, nearly all studies from the global South regions (Africa, Asia and the Middle East) were quantitative surveys, that is, questionnaire or interviews, except one qualitative semi-structured interview study from Malaysia.^32^

### Overall quality of studies

A quality assessment and comparison of the global ICROMS minimum score requirements for each included study is presented in Supplemental Material 2.^25^ Of all 80 studies, 75 met the ICROMS minimum score requirement, 36 did not meet one mandatory criteria and 4 did not meet two mandatory criteria. This suggests that half of the studies were of low quality. For the 27 qualitative studies identified, ICROMS global quality scores ranged from 14 to 23 (mean = 20, ICROMS minimum score requirement = 16). Another nine qualitative studies did not pass the mandatory criteria for the sampling dimension (2F), although their overall score met the minimum score requirement.

For the 40 survey studies identified, the ICROMS minimum scores ranged from 13 to 22 (mean = 18.6, ICROMS minimum score requirement (based on the criteria for qualitative studies) = 16). From this group, three studies^33^–^35^ did not pass the minimum score requirement. The ICROMS global scores for the 11 mixed methods studies ranged from 17 to 21 (mean = 19.6, ICROMS minimum score requirement (based on the criteria for qualitative studies) = 16), and all met the minimum score requirement. The one controlled before-after study^36^ had a quality score of 23, passing the minimum ICROMS score requirement of 18. However, it did not pass the mandatory criteria for the sampling dimension (2D). The one cohort study^30^ had a quality score of 17 which missed the ICROMS minimum requirement of 18. The main issue encountered for most studies was due to managing bias in sampling or between groups, and establishing clear aims and justification, that is, providing a definitive explanation of the study design and specifying the rationale for the choice of research method.

### Data synthesis of included studies

A summary of our included studies is presented in Table 4. The study findings were synthesised to identify themes, informed by the review’s objectives, on how the physical and social aspects of the community pharmacies may influence engagement and satisfaction with pharmacy health services and possible health and/or social or professional implications of these. Most studies explored the theme relating to ‘privacy’, followed by ‘experience of the physical environment’, while fewer explored ‘professional image’ and ‘risk of error’. Some studies included more than one theme and some themes overlapped, particularly aspects relating to privacy and experience of the physical environment.

**Table 4:** Summary of included studies

Study and author(s)	Country	Study design	Characteristics of participants, pharmacy site and any specified health conditions or services (sample size)^a^	Physical and social aspects of community pharmacy space investigated^b^
*Cohort study*				
Marques et al.^30^	The UK	Mixed methods	Pharmacists (209)	Perspectives on organisational culture in the pharmacy environment
*Controlled before-after study*				
Mobach^36^	The Netherlands	Experimental study	Patients (800) and community pharmacies (2)	Visual and acoustic privacy, being observed and overhearing conversations
*Qualitative studies*				
Allan et al.^37^	Scotland	In-depth interviews	Smokers (14)	Privacy within the pharmacy environment
Aradottir and Kinnear^38^	Scotland	Focus group	Pharmacists (4) and gastrointestinal conditions (dyspepsia)	Privacy within the pharmacy environment
Cassie et al.^39^	Scotland	Semi-structured interviews	Pharmacists (19) and medicine counter assistants (11)	Privacy within the pharmacy environment
Chui et al.^40^	The US	Semi-structured interviews	Pharmacists (8) and community pharmacies (6)	Consultation area
Crawford et al.^41^	The US	Semi-structured interviews	Pharmacists (6) and homosexual men (8)	Privacy within the pharmacy environment
DaCosta et al.^42^	England	Semi-structured interviews	Pharmacists (16) and stroke survivor patients (15)	Consultation area
Donovan and Paudyal^43^	England	Semi-structured face-to-face interviews	Pharmacy support staff (21) and community pharmacies (21) (independent pharmacies (9) and chains (12))	Consultation area
Gidman and Coomber^44^	Scotland	Focus groups	Pharmacy service users (26) and opioid substitution therapy services	Perspectives on open plan pharmacy spaces and privacy
Gray et al.^45^	New Zealand	Semi-structured face-to-face interview	Pharmacists (11); community pharmacies (11); and weight management service	Consultation area
Hattingh et al.^46^	Australia	In-depth interviews	Pharmacy service users and carers (74) and mental health conditions (depression, anxiety, bipolar affective disorder and schizophrenia and other psychotic disorder)	Privacy within the pharmacy environment
Hattingh et al.^47^	Australia	Open-ended face-to-face interviews	Pharmacists (25); pharmacy service users (55); and community pharmacies (25) (independent pharmacies (13) and chains (12))	Privacy within the pharmacy environment
Kho et al.^32^	Malaysia	Semi-structured interviews	Pharmacists (20); community pharmacies (20) (independent (14) and chains (6)); and location: city (15) and rural (5)	Lack of space in pharmacy
Lawrie et al.^48^	Scotland	Semi-structured interviews	Community pharmacies (10); pharmacy service users (80); and drug misuse services	Privacy within the pharmacy environment
McMillan et al.^49^	Australia	Semi-structured interviews	Pharmacists (11); younger pharmacy service users (aged 14–25 years); and mental health conditions (18)	Privacy within the pharmacy environment
Mobach^50^	The Netherlands	Interviews	Pharmacists (8) and community pharmacies (8)	Consultation area
Norris and Rowsell^51^	New Zealand	Qualitative analysis of written accounts	Pharmacy service users (12) and community pharmacies (180)	Privacy within the pharmacy environment
Le and Braunack-Mayer^52^	Australia	Semi-structured face-to-face interviews	Community pharmacies (1) and opioid substitution treatment patients (14)	Privacy within the pharmacy environment
Pumtong et al.^53^	England	Semi-structured face-to-face interviews	Pharmacists (26); community pharmacies (25) (independent pharmacies (14) and chains (11))	Privacy within the pharmacy environment
Rapport et al.^54^	England	Consultation workshops using biophotographic data	Pharmacists (24); pharmacy support staff (4); and pharmacy service users (6)	Privacy and professional image within the pharmacy environment
Saramunee et al.^55^	England	Focus groups	Pharmacists (9) and pharmacy-based public health services	Consultation area
Seubert et al.^56^	Australia	Focus groups	Pharmacists (28); pharmacy assistants (5); and pharmacy service users (27)	Privacy within the pharmacy environment
Steckowych et al.^57^	The US	Focus groups	Pharmacy service users (18) and community pharmacies (18) (independent pharmacies (1) and chains (17))	Privacy within the pharmacy environment
Thompson and Bidwell^58^	New Zealand	Focus groups	Pharmacists (20) and pharmacy service users (27)	Professional image
Tucker and Stewart^59^	England	Semi-structured telephone interviews	Community pharmacies (7); patients (25); location: city (2), suburbs (3) and rural (2); and skin conditions	Privacy within the pharmacy environment
Watson et al.^60^	The UK	Focus groups and interviews	Pharmacy service users (20)	Privacy within the pharmacy environment
Wilkinson et al.^61^	The US	Semi-structured telephone interviews	Older teens (aged 18–19) (30) and birth control services	Privacy within the pharmacy environment
Wood et al.^62^	Australia	Semi-structured interviews	Pharmacists (12) and sexual health services	Consultation area
*Survey studies*				
Akram et al.^63^	Malaysia	Self-administered questionnaire	Pharmacists (150); community pharmacies (150) (independent (26) and chains (124)); location in cities (150); and asthma management services	Consultation area
Al-Arifi^64^	Saudi Arabia	Self-administrative questionnaire	Pharmacists (43); community pharmacies (9); and mental health services (schizophrenia, depression, mania, paranoia, panic, obsessive compulsive disorder (OCD) and anxiety)	Consultation area
Saad Ali et al.^65^	The United Arab Emirates	Self-administered questionnaire	Patients (210)	Privacy within the pharmacy environment
Al Laif et al.^66^	Saudi Arabia	Questionnaire	Community pharmacists (58)	Privacy within the pharmacy environment
Allison et al.^67^	England	Questionnaire	Community pharmacies (77) and heart disease screening	Privacy and lack of space within the pharmacy environment
Alsaleh et al.^68^	Kuwait	Self-administered questionnaire	Pharmacists (253)	Safety culture within the pharmacy environment
Alotaibi and Abdelkarim^33^	Saudi Arabia	Structured face-to-face questionnaire	Pharmacy service users (100)	Privacy within the pharmacy environment
Alsabbagh et al.^69^	Canada	Questionnaire	Community pharmacies (6); pharmacy service users (541); and influenza vaccinations	Privacy within the pharmacy environment
Barnard et al.^70^	The US	Questionnaire	Female pharmacy service users (60) and intimate partner violence (IPV)	Lack of comfortable space in the pharmacy
Bawazir^71^	Saudi Arabia	Self-administered questionnaire	Pharmacy service users (911) and community pharmacies (55)	Privacy within the pharmacy environment
Cagirci et al.^72^	Turkey	Face-to-face interviews	Pharmacists (200) and community pharmacies (200)	Physical appearance of the pharmacy
Castaldo et al.^73^	Italy	Telephone interviews	Pharmacy service users (735)	Physical appearance and layout of the pharmacy
Domiati et al.^74^	Lebanon	Self-administered questionnaire	Pharmacists (820)	Consultation area
El-Sharif et al.^75^	The United Arab Emirates	Questionnaire	Patients (375)	Privacy within the pharmacy environment (consultation area)
Ghattas and Al-Abdallah^76^	Jordan	Self-administered questionnaire	Pharmacy service users (801)	Physical pharmacy environment not considered important
Hall et al.^77^	Australia	Semi-structured questionnaires	Pharmacy service users (537) and mental health conditions (depression, anxiety, post-traumatic stress disorder (PTSD), bipolar disorder, OCD, panic attacks and schizophrenia)	Privacy within the pharmacy environment
Iskandar et al.^78^	Lebanon	Questionnaire	Patients (565) and community pharmacies (42)	Privacy within the pharmacy environment
Khdour and Hallak^79^	Palestine	Questionnaire	Pharmacy service users (790) and community pharmacies (39)	Privacy within the pharmacy environment
Knowles et al.^80^	England	Questionnaire	Pharmacists (263) and community pharmacies (263)	Consultation area
Krska and Morecroft^81^	England	Questionnaire	Healthy adult pharmacy service users (300) and public health role of community pharmacies	Privacy within the pharmacy environment
Laird et al.^82^	Scotland	Semi-structured questionnaires	Pharmacists (508); community pharmacies (111) (independent (43) and chain or health centre (67)); location (cites (108) and suburbs (2)); and drug misuse services	Privacy within the pharmacy environment
Lea et al.^83^	Australia	Self-administered questionnaire	Pharmacy service users (508); community pharmacies (50); and opioid treatment services	Privacy within the pharmacy environment
Liekens et al.^84^	Belgium	Questionnaire	Pharmacists (149) and mental health (depression)	Privacy within the pharmacy environment
Malewski et al.^85^	The US	Self-administered questionnaire	Patients (326)	Privacy within the pharmacy environment
Mamen et al.^86^	Norway	Questionnaire	Older pharmacy service users (162), (age ⩾65 years)	Privacy within the pharmacy environment
Mehralian et al.^87^	Iran	Self-administered questionnaire	Pharmacy service users (797) and community pharmacies (200)	Physical pharmacy environment not considered important
Mohamed et al.^88^	Sudan	Self-administered questionnaire	Pharmacists (183)	Lack of space in the pharmacy
Offu et al.^89^	Nigeria	Questionnaire	Pharmacists (40); community pharmacies (40); public health role of community pharmacies	Lack of space in the pharmacy
Okai et al.^90^	Ghana	Questionnaire	Pharmacy service users (497)	Privacy within the pharmacy environment
Okonta et al.^91^	Nigeria	Semi-administered questionnaire	Pharmacists (19)	Consultation area
Pronk et al.^34^	The Netherlands	Questionnaire	Pharmacists (118)	Lack of space in the pharmacy
Puspitasari et al.^92^	Australia	Self-administered questionnaire)	Pharmacists (209); community pharmacies (209); and cardiovascular disease services	Perspectives on organisational culture in the pharmacy environment
Szeinbach et al.^93^	The US	Questionnaire	Pharmacists (398) and community pharmacies (398) (independent (94) and chain (304))	Risks of error
Teinila et al.^94^	Finland	Open-ended question and Likert-type statements	Pharmacists (340) and community pharmacies (340)	Risks of error
Son et al.^95^	South Korea	Self-completed web-based questionnaire	Members of the public (current or future pharmacy service users) (1000)	Consultation area
Ung et al.^96^	Macao	Questionnaire	Pharmacists (100) and community pharmacies (100) (independent (30) and chain (70))	Consultation area
Villako and Raal^35^	Estonia	Questionnaire	Pharmacists (135); pharmacy service users (1979); community pharmacies (7); location: cities (3), suburbs (2) and rural (2)	Privacy and comfort
Whelan et al.^97^	Canada	Self-administered questionnaire	Pharmacists (451) and contraceptive services	Privacy within the pharmacy environment
Wirth et al.^98^	Malta	Self-administered questionnaire	Pharmacy service users (500) and community pharmacies (50)	Privacy within the pharmacy environment
Xi et al.^99^	China	Questionnaire	Pharmacists (163) and community pharmacies (163) (independent pharmacies (74) and chains (89))	Lack of privacy and space
*Mixed methods studies*				
Angelo et al.^100^	The US	Survey and observation	Pharmacists (11); patients (173); and chain community pharmacies (4)	Privacy within the pharmacy environment
Deeks et al.^101^	Australia	Questionnaire and focus groups	Pharmacy assistants (36); community pharmacies (6); location: cities (4) and suburbs (2); and sexual health services	Privacy within the pharmacy environment
Hattingh et al.^102^	Australia	Surveys and semi-structured interviews	Pharmacists (142); pharmacy support staff (21); community pharmacies (100); and mental health services (depression and anxiety)	Privacy within the pharmacy environment
Horsfield et al.^103^	New Zealand	Survey and qualitative consultation	Pharmacists (251); young people (aged 12–25 years) (8); community pharmacies (251) (independent (129) and chains (117)); and location: cities (191) and rural (54)	Privacy within the pharmacy environment
Horvat and Kos^104^	Slovenia	Semi-structured interviews and Delphi technique	Patients (43)	Privacy and working environment within the pharmacy
Munro et al.^105^	England	Survey and face-to-face interviews	Not possible to determine sample size of participants who reported about the pharmacy environment	Privacy within the pharmacy environment
O’Reilly et al.^106^	Australia	Semi-structured interviews	Pharmacists (20); community pharmacies (12) (independent (8) and chain (4); location: cities (9) and rural (3); and mental health (depression) screening services	Consultation area and professional image
Pumtong et al.^107^	England	Semi-structured interviews and survey	Not possible to determine sample size of participants who reported about the pharmacy environment	Privacy within the pharmacy environment
Rapport et al.^108^	Wales	Qualitative biophotographic study	Pharmacists (16); community pharmacies (16) (independent (5) and chains (11))	Perspectives on pharmacy spaces (dispensary, consultation room and sales area) and professional image
Rapport et al.^109^	Wales	Consultation workshops by bio-photographic data	Pharmacists (16); community pharmacies (16) (independent (5) and chains (11)) Same data as Rapport et al.^108^	Lack of privacy and space
Rogers et al.^110^	England	Observation and telephone interviews	Pharmacy service users (44); community pharmacies (10) (independent (5) and chains (5)); location: cities (6), suburbs (2) and rural (2); and perceptions of advise giving services	Consultation area

a For some studies, the sample size presented here relate only to part of the study which explored pharmacy spaces.

b Some physical and social aspects of the community pharmacy overlapped; all these are not detailed in the summary table, that is, when consultation areas were mentioned, participants also expressed concerns about privacy.

#### Privacy

Privacy was a major theme reported in 51 studies, which demonstrate the significance of this issue. Participants were most dissatisfied with lack of privacy,^39,56,78,81,98,104,105,107^ and small pharmacy spaces.^46,47,51^ In some regions, pharmacies did not have a separate consultation area.^45,61,63^ Having a private consultation room or a dedicated private area was considered important,^33,60,98^ as this allowed participants to have confidential conversations with the pharmacist.^50^

Privacy was of concern during patient medication reviews which took place in pharmacies without a consultation room, such as in Norway^86^ and Lebanon.^74^ Lack of privacy was thought to affect participants’ behaviour, including reduced understanding about treatment during over-the-counter medication counselling.^56^ Privacy was a factor for participants when choosing a particular pharmacy for opioid substitution therapy,^44^ favouring those which could offer a private room.^111^ For sexual health, teenagers reported feeling embarrassed to discuss birth control medication in the pharmacy.^61^In addition, participants were concerned about the lack of a comfortable space to have intimate partner violence screening consultations,^70^ and homosexual men reported the lack of a safe place for sexual health screening.^41^

Pharmacists reported a lack of privacy when consulting with patients about their mental health.^77,84,102^ Young mental health patients described a lack of privacy due to pharmacies’ open plan spaces^49^ which was exacerbated during busy periods.^37^ Studies from England and New Zealand showed many pharmacy users were unaware of the presence of a private consultation area,^54,55^ especially young people^103^ and because sometimes pharmacists did not offer the consultation room to their patients.^58,62,71,79^ Privacy problems were also reported when consulting about skin disease,^59^ weight management^45^ and influenza vaccinations.^69^

Alternative views were also expressed. For example, in the United Arab Emirates (UAE), more than a third of participants reported that their privacy had been respected despite the lack of a private consultation room.^75^ Overall, there was an insufficient level of privacy within pharmacy services.^57^ Participants expressed increased privacy could be achieved by reducing noise, moving to a quieter area of the store or avoiding conversations in front of other people.^51^ The dominance of privacy as an issue in pharmacy health service is an important one affecting an individual’s decision to use community pharmacy as the first point of contact.^90^

#### Experience of the physical environment

This broad theme relates to participants’ experiences arising through engaging with the physical environment of pharmacy spaces, which was reported in 39 studies. The physical environment encompasses a range of attributes including space layout, comfort, ease of orientation around the pharmacy, display of merchandise and level of tidiness. These factors are thought to affect pharmacy users’ satisfaction, trust in community pharmacists and loyalty towards the pharmacy.^73^ Convenience and cleanliness of the pharmacy space was found to influence users’ satisfaction.^87^ In contrast, the factor which least affected Jordanian patients’ choice of pharmacy was its physical environment.^76^ This may have been due to the short duration of time spent by patients in pharmacies; thus, the physical space was not considered as important as other issues.

Findings from Rapport *et al.*^108,109^ showed the dispensary to be a space people can look into, giving employees a sense of being monitored and making them susceptible to interruption which overlaps with the theme ‘risk of error’. Some findings suggested consultation rooms should be close to the pickup window, where patients receive their prescription, the space should have computer access, comfortable seating, a whiteboard and easy access for disabled people.^40,60^ Pharmacists also preferred to consult in a quiet area, separate from the counter.^80^ There was also accessibility problem for stroke survivors in wheelchairs and caregivers,^42^ and it was recommended the consultation rooms should be larger to reduce discomfort.^109^ Some viewed these spaces as undesirable if it was used by patients receiving treatment for drug problems.^55,110^ In Scotland, patients in treatment for drug problems were reluctant to use these rooms as they feared being identified as a ‘methadone client’,^44^ and perceived it to be an uncomfortable or embarrassing space.^39^

Findings relating to experience of the physical environment was also connected to the pharmacy waiting area. Unsurprisingly, given the typical size of a community pharmacy, the waiting area was described as being small.^110^ Some respondents indicated that having a seat improved comfort, and information on the wall was useful while awaiting HIV screening results.^41^ Likewise, a survey study showed that comfortable waiting areas in Tehran enhanced patients’ satisfaction.^87^ However, in an urban pharmacy sales area, glass partitions with shelves filled with items obstructed the pharmacist’s view of patients in the waiting area.^110^

#### Professional image

Four studies addressed this theme. The design of open spaces influenced pharmacists’ sense of self-worth and professionalism, and the orderliness of the environment affected the way patients perceived pharmacy staff level of professionalism.^109^ The same issue applied to the dispensary, where this space is shared with other staff for a prescription preparation or checking. A tidy dispensary made the space look more professional and less stressful.^108,109^ Ideally, the pharmacy counter should be a safe space which reflects the professional identity of the pharmacist and the store.^58^ It was found that spaces were not always used for their designated function. Consultation rooms were sometimes used as a temporary storage room, which detracted from the professional image.^108^ The relationship between a sales area and a pharmacy counter was interesting: large chain stores sometimes have no clear boundaries between these areas, requiring the pharmacist to act as a salesperson at the same time. This, too, may be perceived as unprofessional by pharmacy patients.^54^

#### Risk of error

Only three studies addressed this theme. Pharmacists reported that poor design of the physical space (e.g. work area, storage and shelving) contributed to dispensing errors and difficulties with communicating with other staff.^93^ Another survey found that the working environment (e.g. space, equipment and noise) causes dispensing errors which could be prevented by a well-designed workspace.^94^ Pharmacists perceived an environment that is well organised and free of clutter, and whose physical layout supports good workflow would be conducive to achieve high patient safety standards.^68^ Pharmacists in Finland reported that the most likely cause of dispensing errors was a lack of dispensary work space.^94^ It is clear that structured planning in this area could help prevent dispensing errors.

## Discussion

This is the first known comprehensive review to systematically examine published research on how community pharmacy spaces are experienced by pharmacy service users and staff. From searching 4517 publications, we identified 80 papers which described 80 studies, published between 1994 and 2020, from 28 countries across six continents and region. Studies used a range of designs, including surveys, interviews, focus groups and mixed methods approaches. There were a diverse range of health conditions included in the studies: drug and alcohol problems; mental health; sexual health; heart disease; gastrointestinal conditions; respiratory disease; skin conditions; and weight management. Such diversity highlights the variety of pharmacy health services offered across the globe and signifies community pharmacy to be vital space for public health. Although half of the studies did not meet the ICROMS mandatory quality criteria, the majority met the minimum quality score (94%). The studies were largely explorative in nature, thus highlighting how research evidence on optimal pharmacy design is still lacking.

Half the studies were mixed methods or qualitative in design, and the exploratory nature of the study designs may have enabled participants to express ideas about pharmacy spaces more readily, especially during qualitative interviews, even if space was not the primary focus. The data synthesis enabled the establishment of four overall themes, ‘privacy’; ‘experience of the physical environment’; ‘professional image’; and ‘risk of error’. The review highlighted the importance of the pharmacy design. Factors influencing pharmacy users’ level of comfort included size, structure and design of the pharmacy space. From the staff perspective, the pharmacy layout influenced their sense of professionalism. The lack of privacy and space were two main environmental factors that affected pharmacy users and staff engagement. In addition, there was some misunderstanding of the purpose of the consultation room, for example, it was assumed to be used solely for the provision of particular pharmaceutical services such as drug misuse treatments.

Reasons for the scarcity of research about the impact of pharmacy spaces on healthcare outcomes is an interesting question which warrants further investigation. One possible explanation could be the slow development of interdisciplinary pharmacy practice education and research.^112^ It is relatively recently that psychosocial community pharmacy health service research has gained ground, particularly in the global North, with advancement of new professional roles for pharmacists including independent prescribing, medication optimisation and other public health services.^5,8^ Research during this period examined pharmacists’ communication skills and patient’s health outcomes; however, very few focussed on sensory or the visual experience of space,^54,58,108,109^ and there is a conspicuous lack of studies informed by the arts and health architecture theories. This may explain the lack of findings exploring sensory experience in the review studies. A lack of interdisciplinary thinking within pharmacy practice research may be one reason for the absence of review studies examining pharmacy spaces. In addition, pharmacy practice research may not yet have attracted diverse individuals from a range of disciplines to share knowledge and experience. Pharmacy education and profession have traditionally been viewed as a science,^113^ whereas medical education, health and social care training have evolved to embed the humanities and the arts, with some promising outcomes for patients, practitioners and students.^114,115^ It will be interesting to see if and how pharmacy practice could integrate interdisciplinary thinking, especially the arts and participatory co-design approaches;^116,117^ particularly to effectively optimise pharmacy spaces to improve health and wellbeing. To understand the broader spectrum of wellness and illness, application of salutogenic architecture could yield valuable insights for pharmacy.^12^ Such interdisciplinary enquiry could accelerate pharmacy research in new directions, and have important implications for public health, particularly to further realise the potential impact of pharmacy as a key point of contact for health globally.

### Strengths and limitations

This is the first comprehensive review to systematically examine the published research on how community pharmacy spaces are experienced by pharmacy service users and staff. The findings reported are from a range of continents, which adds to its strength; however, it is not possible to generalise the findings across such diverse communities as these could be culture specific, that is, different meanings could be attached to ‘pharmacy space’.

### Future directions

Future research could focus on pharmacy service users’ and staff experiences of pharmacy spaces as its primary aim and examine the potential benefit of inclusive pharmacy design features which specifically address sensory experience of space. In addition, privacy; professional image; and reducing risk from practice errors could be explored to examine implications of these for different cultures and communities. A participatory co-design approach could helpfully identify optimal designs which could then be evaluated prospectively in terms of impact on health outcomes, and both service user and staff outcomes.

## Supplemental Material

sj-docx-1-rsh-10.1177_17579139221080608 – Supplemental material for Systematic review on the effects of the physical and social aspects of community pharmacy spaces on service users and staff Click here for additional data file. Supplemental material, sj-docx-1-rsh-10.1177_17579139221080608 for Systematic review on the effects of the physical and social aspects of community pharmacy spaces on service users and staff by R Dhital, S Sakulwach, G Robert, C Vasilikou and J Sin in Perspectives in Public Health

sj-docx-2-rsh-10.1177_17579139221080608 – Supplemental material for Systematic review on the effects of the physical and social aspects of community pharmacy spaces on service users and staff Click here for additional data file. Supplemental material, sj-docx-2-rsh-10.1177_17579139221080608 for Systematic review on the effects of the physical and social aspects of community pharmacy spaces on service users and staff by R Dhital, S Sakulwach, G Robert, C Vasilikou and J Sin in Perspectives in Public Health
