# Supplementary material for: Systematic review on the effects of the physical and social aspects of community pharmacy spaces on service users and staff
Source: Perspect Public Health. 2022 Mar 11;142(2):77–93. doi: 10.1177/17579139221080608 (PMC8918882; doi:10.1177/17579139221080608)
Supplement: sj-docx-1-rsh-10.1177_17579139221080608 – Supplemental material for Systematic review on the effects of the physical and social aspects of community pharmacy spaces on service users and staff [file sj-docx-1-rsh-10.1177_17579139221080608.docx]

**Supplementary Material 1:**

**Search Terms and Results**

PubMed <1990 to Present>

Search Strategy:

1. Pharmacy

(“community pharmacy” or “community pharmacies” or pharmacy or pharmacies) < 48,301>

2. Pharmacy environment

(“pharmacy design” or “interior design” or “evidence-based design” or physical environment* or social environment* or architecture* or workspace* or space or lighting* or noise* or privacy or “work station” or people flow* or safety* environment* or security* or comfort* environment* or “centre of built environment”) <100,502>

3. Pharmacy outcomes

(perception* or experience* or satisfact* or participat* or observation* or impression* or emotional effect* or environment effect* or engagement* or involve* or attitude* or “work efficiency” or performance* or “work flow” or work productivity or team work) < 4,011,634>

4. Pharmacy staffs

(pharmacist* or chemist* or “counter staff” or technician*) <219,819>

5. Pharmacy patients

(user* or “service user*” or customer* or patient* or client*) <6,737,765>

6. 4 or 5 <6,924,237>

7. 1 and 2 and 3 < 188> / 1 and 2 <365> *To select without pharmacy outcomes

8. 6 and 7 <150> / <273>

9. limit and filter 8 to (languages: (english) and humans)

<98> / <179>
