# Supplementary material for: Systematic review on the effects of the physical and social aspects of community pharmacy spaces on service users and staff
Source: Perspect Public Health. 2022 Mar 11;142(2):77–93. doi: 10.1177/17579139221080608 (PMC8918882; doi:10.1177/17579139221080608)
Supplement: sj-docx-2-rsh-10.1177_17579139221080608 – Supplemental material for Systematic review on the effects of the physical and social aspects of community pharmacy spaces on service users and staff [file sj-docx-2-rsh-10.1177_17579139221080608.docx]

**Supplementary Material 2:**

Quality assessment of included studies using ICROMS

| Study design category, ICROM dimension scores presented as **(maximum possible score)** and (actual score) | Aims and justification | Sampling | Outcome measure | Follow-up | Other study aspects | Analytical rigour | Other considerations | Total score |
| --- | --- | --- | --- | --- | --- | --- | --- | --- |
| **Cohort study (18)*** | **(2)** | **(2)** | **(6)** | **(2)** | **(2)** | **(2)** | **(10)** | **(26)** |
| Marques et al., 2018 | 2 | 0 | 5 | 2 | 0 | 1 | 7 | 17 |
| **Controlled before-after study (18)*** | **(2)** | **(2)** | **(8)** | **(2)** | **(2)** | **(2)** | **(10)** | **(28)** |
| Mobach M. P., 2009 | 2 | 1 | 8 | 2 | 2 | 2 | 6 | 23 |
| **Qualitative study (16)*** | **(6)** | **(2)** | **(2)** | **(2)** | **(2)** | **(2)** | **(10)** | **(26)** |
| Allan et al., 2012 | 5 | 1 | 2 | 2 | 1 | 1 | 8 | 20 |
| Aradottir & Kinnear, 2008 | 4 | 1 | 0 | 1 | 1 | 1 | 6 | 14 |
| Cassie et al., 2019 | 5 | 2 | 2 | 2 | 2 | 2 | 8 | 23 |
| Chui et al., 2012 | 5 | 2 | 2 | 2 | 1 | 1 | 9 | 22 |
| Crawford et al., 2020 | 5 | 2 | 2 | 2 | 1 | 1 | 9 | 22 |
| DaCosta et al., 2019 | 5 | 2 | 2 | 2 | 1 | 1 | 9 | 22 |
| Donovan & Paudyal, 2016 | 5 | 2 | 2 | 2 | 1 | 1 | 9 | 22 |
| Gidman & Coomber, 2014 | 5 | 2 | 2 | 2 | 1 | 1 | 9 | 22 |
| Gray et al., 2016 | 5 | 1 | 2 | 2 | 1 | 1 | 7 | 19 |
| Hattingh et al., 2015 | 5 | 2 | 2 | 1 | 2 | 1 | 9 | 22 |
| Hattingh et al., 2016 | 5 | 2 | 2 | 2 | 1 | 1 | 8 | 21 |
| Kho et al., 2017 | 5 | 2 | 2 | 2 | 1 | 1 | 8 | 21 |
| Lawrie et al., 2004 | 5 | 1 | 2 | 2 | 1 | 1 | 7 | 19 |
| McMillan et al., 2020 | 5 | 1 | 2 | 2 | 1 | 1 | 8 | 20 |
| Mobach M. P., 2005 | 5 | 1 | 2 | 2 | 1 | 1 | 5 | 17 |
| Norris & Rowsell, 2003 | 5 | 1 | 1 | 1 | 1 | 1 | 6 | 16 |
| Phuong-Phi & Braunack-Mayer, 2019 | 5 | 2 | 2 | 2 | 1 | 2 | 9 | 23 |
| Pumtong et al., 2008 | 5 | 2 | 0 | 2 | 1 | 1 | 7 | 18 |
| Rapport et al., 2010 | 5 | 1 | 2 | 2 | 1 | 1 | 7 | 19 |
| Saramunee et al., 2014 | 5 | 1 | 0 | 2 | 1 | 1 | 8 | 18 |
| Seubert et al., 2017 | 5 | 2 | 2 | 2 | 1 | 1 | 8 | 21 |
| Steckowych et al., 2019 | 5 | 2 | 2 | 2 | 1 | 1 | 7 | 20 |
| Thompson & Bidwell, 2015 | 5 | 2 | 2 | 1 | 1 | 1 | 8 | 20 |
| Tucker & Stewart, 2015 | 5 | 2 | 1 | 2 | 1 | 1 | 8 | 20 |
| Watson et al., 2019 | 5 | 2 | 2 | 2 | 1 | 1 | 6 | 19 |
| Wilkinson et al., 2018 | 5 | 2 | 2 | 1 | 1 | 1 | 8 | 20 |
| Wood et al., 2018 | 5 | 1 | 2 | 2 | 1 | 2 | 8 | 21 |
| **Survey study (16)*** | **(2)** | **(2)** | **(8)** | **(2)** | **(2)** | **(2)** | **(10)** | **(28)** |
| Akram et al., 2018 | 4 | 2 | 0 | 2 | 1 | 1 | 7 | 17 |
| Al-Arifi M. N., 2008 | 5 | 1 | 2 | 2 | 1 | 1 | 9 | 21 |
| Saad Ali et al., 2019 | 5 | 1 | 2 | 2 | 1 | 1 | 8 | 20 |
| Al Laif et al., 2017 | 5 | 2 | 0 | 2 | 1 | 1 | 7 | 18 |
| Allison et al., 1994 | 5 | 2 | 0 | 2 | 1 | 1 | 5 | 16 |
| Alsaleh et al., 2018 | 5 | 2 | 2 | 2 | 1 | 1 | 9 | 22 |
| Alotaibi & Abdelkarim, 2015 | 5 | 1 | 2 | 1 | 1 | 0 | 5 | 15 |
| Alsabbagh et al., 2019 | 5 | 1 | 0 | 2 | 1 | 1 | 9 | 19 |
| Barnard et al., 2018 | 4 | 2 | 2 | 2 | 1 | 1 | 8 | 20 |
| Bawazir S. A., 2004 | 5 | 2 | 2 | 2 | 1 | 1 | 5 | 18 |
| Cagirci et al., 2012 | 5 | 2 | 0 | 2 | 1 | 1 | 8 | 19 |
| Castaldo et al., 2016 | 5 | 2 | 2 | 2 | 1 | 1 | 6 | 19 |
| Domiati et al.., 2018 | 5 | 2 | 2 | 2 | 1 | 1 | 8 | 21 |
| El-Sharif et al., 2017 | 5 | 1 | 2 | 2 | 1 | 1 | 6 | 18 |
| Ghattas & Al-Abdallah, 2020 | 5 | 2 | 2 | 2 | 1 | 1 | 7 | 20 |
| Hall et al., 2019 | 5 | 1 | 0 | 2 | 1 | 1 | 8 | 18 |
| Iskandar et al., 2017 | 5 | 2 | 0 | 2 | 1 | 1 | 9 | 20 |
| Khdour & Hallak, 2012 | 5 | 2 | 2 | 2 | 1 | 1 | 8 | 21 |
| Knowles et al., 2002 | 5 | 2 | 0 | 2 | 1 | 1 | 7 | 18 |
| Krska & Morecroft, 2010 | 4 | 1 | 0 | 2 | 1 | 1 | 8 | 17 |
| Laird et al., 2016 | 5 | 1 | 2 | 2 | 1 | 1 | 7 | 19 |
| Lea et al., 2008 | 5 | 1 | 2 | 2 | 1 | 1 | 7 | 19 |
| Liekens et al., 2012 | 4 | 2 | 0 | 2 | 1 | 1 | 7 | 17 |
| Malewski et al., 2015 | 4 | 1 | 0 | 2 | 1 | 1 | 8 | 17 |
| Mamen et al., 2015 | 5 | 1 | 2 | 2 | 1 | 1 | 9 | 21 |
| Mehralian et al., 2014 | 5 | 1 | 2 | 2 | 1 | 1 | 7 | 19 |
| Mohamed et al., 2014 | 5 | 2 | 0 | 2 | 1 | 1 | 8 | 19 |
| Offu et al., 2015 | 5 | 2 | 2 | 2 | 1 | 1 | 8 | 21 |
| Okai et al., 2020 | 5 | 2 | 2 | 2 | 1 | 1 | 7 | 20 |
| Okonta et al., 2012 | 4 | 1 | 2 | 2 | 1 | 1 | 5 | 16 |
| Pronk et al., 2002 | 5 | 1 | 0 | 1 | 1 | 1 | 5 | 14 |
| Puspitasari et al., 2016 | 5 | 2 | 0 | 2 | 1 | 1 | 9 | 20 |
| Szeinbach et al., 2007 | 5 | 1 | 2 | 2 | 1 | 1 | 7 | 19 |
| Teinila et al., 2008 | 5 | 2 | 2 | 2 | 1 | 1 | 8 | 21 |
| Son et al., 2019 | 5 | 1 | 0 | 2 | 1 | 1 | 8 | 18 |
| Ung et al., 2016 | 5 | 1 | 0 | 2 | 1 | 1 | 8 | 18 |
| Villako & Raal, 2007 | 5 | 1 | 0 | 1 | 1 | 0 | 5 | 13 |
| Whelan et al., 2013 | 5 | 2 | 0 | 2 | 1 | 1 | 7 | 18 |
| Wirth et al., 2010 | 4 | 2 | 0 | 2 | 1 | 1 | 7 | 17 |
| Xi et al., 2019 | 5 | 2 | 1 | 2 | 1 | 1 | 7 | 20 |
| **Mixed-method study (16)*** | **(6)** | **(2)** | **(2)** | **(2)** | **(2)** | **(2)** | **(10)** | **(26)** |
| Angelo et al., 2005 | 5 | 1 | 2 | 2 | 1 | 1 | 8 | 20 |
| Deeks et al., 2014 | 5 | 1 | 2 | 2 | 1 | 1 | 9 | 21 |
| Hattingh et al., 2017 | 5 | 2 | 0 | 2 | 1 | 1 | 9 | 20 |
| Horsfield et al., 2014 | 5 | 1 | 1 | 2 | 1 | 1 | 9 | 20 |
| Horvat & Kos., 2011 | 6 | 2 | 2 | 2 | 1 | 1 | 7 | 21 |
| Munro et al., 2003 | 5 | 2 | 2 | 1 | 1 | 1 | 8 | 20 |
| O’Reilly et al., 2015 | 5 | 1 | 0 | 2 | 1 | 1 | 7 | 17 |
| Pumtong et al., 2011 | 4 | 2 | 2 | 2 | 1 | 1 | 8 | 20 |
| Rapport et al., 2009 (a) | 5 | 2 | 2 | 2 | 1 | 1 | 7 | 20 |
| Rapport et al., 2009 (b) | 5 | 2 | 2 | 2 | 1 | 1 | 7 | 20 |
| Rogers et al., 1998 | 4 | 2 | 2 | 2 | 1 | 1 | 5 | 17 |

Note: *ICROMS minimal score requirement: below minimum score; above minimum score
